# An Overview of Recent Strategies in Pathogen Sensing

**DOI:** 10.3390/s90604483

**Published:** 2009-06-08

**Authors:** Jinseok Heo, Susan Z. Hua

**Affiliations:** 1 Bio-MEMS and Biomaterials Laboratory, Department of Mechanical and Aerospace Engineering, University at Buffalo, The State University of New York, Buffalo, NY 14260, USA; 2 Department of Physiology and Biophysics, University at Buffalo, The State University of New York, Buffalo, NY 14241, USA

**Keywords:** pathogen, microfabrication, nanofabrication, lab-on-a-chip, microfluidics

## Abstract

Pathogenic bacteria are one of the major concerns in food industries and water treatment facilities because of their rapid growth and deleterious effects on human health. The development of fast and accurate detection and identification systems for bacterial strains has long been an important issue to researchers. Although confirmative for the identification of bacteria, conventional methods require time-consuming process involving either the test of characteristic metabolites or cellular reproductive cycles. In this paper, we review recent sensing strategies based on micro- and nano-fabrication technology. These technologies allow for a great improvement of detection limit, therefore, reduce the time required for sample preparation. The paper will be focused on newly developed nano- and micro-scaled biosensors, novel sensing modalities utilizing microfluidic lab-on-a-chip, and array technology for the detection of pathogenic bacteria.

## 1. Introduction

Detection of pathogenic bacteria in food, water, and air has been an important issue for scientists because of its critical impact on public health. Although standard microbiological methods of cell culture and plating are confirmative to identify bacterial strains [1], it often takes several days to complete the processes. In addition, most of conventional methods require intricate instrumentation and cannot be used on-site. Thus, both private and government sectors strongly need biosensors that can detect pathogens in a fast and accurate manner.

Pathogen sensors must meet several requirements. First, they should show high sensitivity and a low detection limit. Since the speed of multiplication of bacteria is very high, even low numbers of bacteria cells (<10 cells) can be a risk to a patient's health [2]. USDA requires zero tolerance of certain strains of bacteria, such as *E. coli* O157:H7, Salmonella, and *L. monocytogenes*, in food products [3,4]. Second, rapid analysis time is essential. This is especially important to take immediate measures for curing victims of pathogens and restricting the spread of pathogens. Third, simultaneous detection and identification of different strains of bacteria is also critical. To achieve this goal, the sensor should show high specificity toward target cells. An array type of sensor displaying independent specificity for multiple targets can be an attractive platform. Fourth, portability and ease-of-use are important for on-site monitoring. In addition, automation can be a significant factor of consideration for a long-term environmental monitoring.

The function of a pathogenic biosensor is to transduce receptor recognition towards the target pathogen into a detectable signal. Pathogenic sensing relies on either immunosensing or nucleic acid detection. Immunosensors are based on the interaction between antigens presented on the target cells and antibodies immobilized on surfaces. The resulting conjugates have been detected via various sensing methods, including fluorescence [5], electrical or electrochemical impedance [5,6], cantilever [7,8], quartz crystalline microbalance (QCM) [2,7], surface plasmon resonance (SPR) [5,7], and magnetoresistivity [9]. Nucleic acid-based sensors detect DNA or RNA originating from target cells. Because cells contain a low copy number of nucleic acids, the sensor generally requires a step of amplifying target nucleic acids using polymerase chain reaction (PCR) or reverse transcriptase PCR (RT-PCR). In addition, there are several intricate strategies for amplifying signals that report the hybridization between probe and target DNA. Using nanoparticles [10] and enzyme labels [11], redox probes [12-14], and intercalators [15] are among those strategies. The target DNA or RNA will also be detected using various physical sensing methods. In general, the ultimate performance of a pathogen sensor relies on the high efficiency of biochemical reactions, high concentration of target analytes, and sensitive detection or transduction methods.

Recent advances in micro- and nano-fabrication technologies have provided unique advantages for developing pathogen sensors in several respects. The sensor probe created with similar or smaller dimensions of a bacterial cell could provide high sensitivity and a low detection limit. Nanoparticles, nanotubes, nanowires, and nanomechanical devices are representative examples used as functional probes for detecting pathogens. In addition, microfabrication technology has made it possible to integrate multiple processes in sequence for one-step sensing or in parallel for high throughput screening.

In this review, we will highlight a group of pathogen sensors developed in the last several years that have taken advantage of advanced micro- and nano-technology. This paper will focus on the principles, features, and advantages of new sensing technologies. We will also describe how the technology could enhance the sensor sensitivity and detection limit.

## 2. Recent Sensing Strategies for Pathogen Detection Based on Microfluidics

One of the main outcomes of microfabrication technology is the creation of microfluidic devices, so called labs-on-a-chip. Microfluidic systems provide a convenient platform for pathogen sensing in both immunosensing and nucleic acid detection. Microfluidic chip devices normally consist of fluid channels and sensing chambers with dimensions of a few to hundreds of microns. Thus, they require minuscule amounts of samples and reagents. The small dimension of microfluidic chips offers high surface to volume ratio which makes it possible to localize target molecules in the sensing zone. In addition, fast mass transport in the microchannel reduces analysis time. Because a microchannel is typically made of glass or plastic, the inner channel surface can be easily functionalized to selectively capture target bacterial cells under continuous flow conditions. This chapter will describe recent efforts of sensing pathogens taking advantages of microfluidic chip.

### 2.1. Label-free bacterial sensor based on electrical and electrochemical detection

Optical, fluorescent, electrical and electrochemical sensing methods are compatible with microfluidic platform. Electrical and electrochemical detection has received attentions, because microelectrodes can be easily fabricated using photolithography and incorporated in a microfluidic channel. In addition, electrical methods do not require a labeling step for sensing target pathogen. This section will focus on recent reports on microfluidic pathogen sensors utilizing electrical or electrochemical detection methods.

#### Impedance based detection

**Figure 1.**
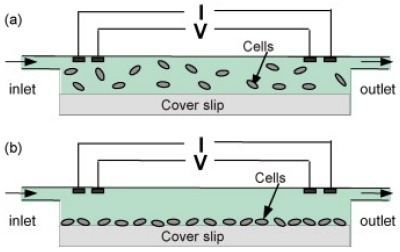
Schematic drawing of impedance based bacteria sensor for suspended (top) and attached cells (bottom). Reproduced with permission from reference [16].

Boehm *et al.* have constructed a microfluidic bacteria sensor based on measuring the impedance in a fixed-volume chamber containing cells [16]. The sensor was microfabricated on silicon chip with thin film platinum electrodes. The measurement chamber was ∼15 μm high and functionalized with antibodies specific to target cells. Bacteria cells in suspension were passed through the chamber so that they could be selectively attached on the modified chamber surface (See Figure 1). Since the membrane of bacterial cells act as an insulator at low alternating current (AC) frequency, the presence of bacteria cells can produce a change in the chamber impedance as they displace an equivalent volume of conducting solution in the chamber. Using this sensor, Boehm *et al.* could discriminate two bacterial strains, *E. coli* and *M.* catarrhalis, in a few minutes. The sensor can detect 9×10^5^ colony forming unit (CFU) mL^-1^ *E. coli cells*. The same group recently demonstrated that the impedance sensor could detect a single mammalian cell by reducing the size of the measurement chamber [17]. It is expected that the detection limit for pathogen detection can be greatly improved by modifying the dimension of the chamber. A similar approach has been used to measure the yeast cell in suspension [18]. In this case, gold thin film was deposited on a small region inside a microfluidic chamber. The gold surface was modified with antibody probes, allowing the attachment of yeast cells. Additionally, impedance across the sensing chamber has been measured to monitor bacteria density, growth, and their long term behavior in response to environmental stimuli [19].

Carbonaro *et al.* have developed an on-chip artificial pore that could be used to detect bacterial pathogens [20]. The microfluidic chip was constructed with polydimethylsiloxane (PDMS) having a fluid channel (a pore) with cross-sectional dimension of 15 × 15 μm. The pore was functionalized with proteins that can specifically interact with cell-surface receptors. Cells suspended in a solution were introduced to the channel. The presence of cells blocks the current across the pore. The target cells that express receptors specific to the immobilized proteins stayed longer inside the pore than control cells. Thus, the duration of the current pulse could discern the difference in the affinity of the cells to the pore surface. The group successfully demonstrated that the artificial pore could screen murine erythroleukemia cells based on their CD34 surface marker.

While the cell membrane is electrically insulating, the intracellular solution is conductive because of the presence of ions. Thus, ions released from cell lysis can lead to a change in the conductivity of solution surrounding cells. Because a total ionic concentration of individual cells does not vary significantly, the extent of change in the solution conductivity after cell lysis can provide the number of lysed cells. This method can be used to count cells. Cheng *et al.* devised a simple microfluidic system consisting of two parallel glass slides and a thin PDMS gasket [21]. After cells were adhered to the glass surface modified with proteins specific to the target cells, they were lysed to monitor a conductivity change. The research group showed the sensor could detect as low as 20 cells/μL. Although the device was developed to count CD 4 cells found in HIV patients, it can be adapted to detect pathogenic bacteria cells.

#### Electrochemical detection

Electrochemical impendence spectroscopy is another label-less sensing technique that is widely used for probing biochemical interactions at the electrodes surface. Since electrochemical impedance sensor detects Faradic current during a redox reaction at the electrode surface, the surface functionality directly affects the sensitivity. Thus, to improve the sensitivity of a pathogen sensor, it is important to optimize surface chemistry for immobilizing antibodies on the electrode surface [22-24]. Liao *et al.* developed a microfluidic electrochemical sensor array for detection of uropathogens in human clinical fluid [25]. The sensor chip consists of sixteen electrochemical cells that can operate independently. The target 16S rRNA was extracted from uropathogenic bacteria present in clinical urine samples and detected using a sandwich hybridization assay. The target RNA was hybridized to a short sequence of capture DNA immobilized on the electrodes and then a reporter DNA conjugated with enzymes. The enzymes were used to produce electroactive species for amperometric detection. The sensor could detect as few as 4 × 10^4^ CFU/mL. Species-specific detection of uropathogenic bacteria could be achieved within 45 min.

#### Interdigitated array electrodes

The electrical impedance output can be further amplified by a parallel set of electrode configuration like interdigitated array (IDA) microelectrodes. An IDA sensor consists of a pair of microcomb array electrodes. A large number of parallel electrodes and a large active surface area improve the detection limit and response time. In addition, IDA can be easily placed in a microfluidic channel using current photolithographic techniques. The attachment of bacteria on the surfaces of an array of electrodes alters both current flow and capacitance between the neighboring electrodes, causing the impedance change in a frequency-dependent manner. Lazcka *et al.* showed that the detection limit of an impedance sensor based on IDA was highly dependent upon electrode geometry and inter-electrodes spacing [26]. As the electrode bands become narrower, the biosensor becomes more sensitive to the presence of bacterial cells. They reported that the electrode bands having 7 μm wide and 13 μm gap could detect bacterial cells as low as 1.50 × 10^3^ cells/mL.

Radke and Alocilja constructed 1,700 lines of IDA based on gold electrodes having a width of 3 μm and an in-between spacing of 4 μm [27]. They tested different concentrations of E. coli. O157:H7 and S. infantis and could detect 10^4^ CFU/mL of E. coli: O157:H7 in 5 minutes. Yang *et al.* also applied IDA microelectrodes for the detection of viable Salmonella Typhimurium in milk samples [28]. The microelectrodes, consisting of indium-tin-oxide (ITO), measure an impedance change during bacterial cell growth. In the initial phase of cell growth the impedance kept stable but later began to decrease. They termed the moment that cells show an impedance decrease as “detection time”. They obtained a linear relationship between the detection time and the logarithmic value of initial cell concentration. A bacterial suspension having the initial concentration of 10^5^ CFU/mL could be detected in 2.2 hours. The detection limit of the sensor could be further enhanced by employing magnetic nanoparticle-antibody conjugates to concentrate the target cells into the sensing region. Varshney *et al.* concentrated target cells using magnetic nanoparticles modified with antibodies and used IDA for sensing. They reported to have detected 1.6 × 10^2^ and 1.2 × 10^3^ cells of E. coli: O157:H7 present in pure culture and ground beef samples, respectively [29].

In contrast to the previous approaches Lu *et al.* have detected bacterial cells (E. coli, JM109) in an insulating environment using IDA gold electrodes [30]. The inter-electrode spacing was further reduced to 2 μm in this device, enabling an attachment of single bacteria across two adjacent electrodes. The attached cells on the electrodes were rinsed with deionized water to remove ions surrounding the cells and dried prior to test. They measured current under dry air condition. The current was closely associated with the number of bacterial cells that formed a conducting bridge between adjacent electrodes. While the sensor is extremely sensitive to detect a single bacterial cell, it requires a clear understanding of conducting mechanism of the bacterial cells on the electrode surface.

IDA was also applied to Faradic impedance sensor that utilizes redox probes on the surface of the electrodes for pathogen detection. Yang *et al.* relied on an IDA system to detect E. coli O157:H7 in milk samples [31]. The IDA consisted of 25 pairs of indium-tin-oxide (ITO) finger electrodes having a dimension of 15 μm wide and 15 μm gap. Yang *et al.* measured electrochemical impedance using [Fe(CN)_6_]^3-/4-^ as a redox probe. Binding of cells to the electrode surfaces generates an impedance change by blocking electron transfer between neighboring electrodes. The detection limit of the sensor was 10^6^ CFU/mL. A detailed review on impedance pathogen sensors can be found in an article recently published [6].

### 2.2. Nucleic acid-based detection

Pathogen sensors based on nucleic acid detection requires several steps, including lysis, extraction of nucleic acids, purification, and detection. In addition, it may be necessary to amplify the number of nucleic acids, because certain nucleic acids are present in a low copy number in the cells. While a lab-on-a-chip sensor can be an attractive platform for conducting the multiple processes, it has been challenging to integrate PCR with other required steps in a chip. This section will cover recent efforts on how to integrate multiple functional modules within a small chip. We will also include new approaches other than immuno- and PCR-based sensing, because neither of the sensing methods can discern the virulence of bacterial cells. Further technical details concerning nucleic acid detection in microfluidic devices can be found in a review paper recently published [32].

#### PCR-based pathogen sensor

PCR is a very promising approach for sensing bacterial pathogens. Theoretically, a single copy of a particular sequence of DNA can be amplified and detected. This technique is highly specific to target cells, because it relies on a primer DNA, which is complementary to a part of the sequence in the bacterial genome. In addition, since PCR can amplify several sets of DNAs simultaneously within a few hours, it can be a useful technique for multiple target detection. The whole steps of concentration, lysis, extraction, purification, and detection has been carried out in a single chip. Microfabricated structures [33], magnetic beads [34], and dielectrophoresis [35-37] are popular strategies to concentrate cells in a microfluidic chip. Then, the bacterial cell wall should be destroyed to extract DNA. Cell lysis can be performed in a chip using various methods, such as thermal energy [38], optothermal energy [39,40], mechanical force [41], and chemicals [42,43]. After DNA extraction, DNA can be purified in a microfluidic chip using packed silica beads [44], microfabricated structures [45], or magnetic beads [38]. Fluorescence and electrochemical methods are most frequently used for DNA detection in a lab-on-a-chip because of ease of miniaturization. In addition, many signaling molecules and nanoparticles are either electroactive or fluorescent.

Laser light can be efficiently converted to the thermal energy in the presence of magnetic particles in a solution. Lee *et al.* showed that the optothermal energy was sufficient to break apart the bacterial cell wall [39]. The use of laser for cell lysis could simplify a chip design without the necessity of incorporating heating elements in the chip. After removing magnetic particles, the microfluidic chip was transferred in a compact real-time PCR system for the amplification and detection of DNA. Their research was extended to use gold nanoparticles. The results show that the gold particles did not attenuate the fluorescence intensity during real-time PCR, suggesting that removal of gold nanoparticles is not necessary [40]. In these reports they could detect *E. coli* at the concentration of 10^4^ cells/μL.

**Figure 2.**
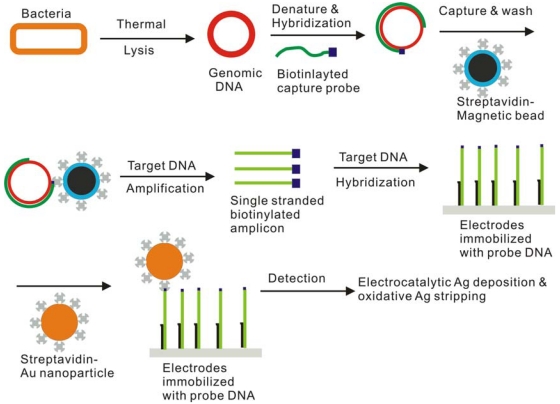
A schematic diagram of pathogenic bacterial detection using microfluidic sensor. Modified with permission from reference [38].

Yeung *et al.* devised a lab-on-a-chip sensor for simultaneously detecting *E. coli* and *B. subtilis* [38] (See also Figure 2). In this work, lysis, PCR, and detection were carried out in a microfluidic device. After thermal lysis, magnetic beads conjugated with capture DNA were used to extract and purify genomic DNA. Then, target DNAs were amplified by PCR. Since probe DNAs were tagged with electroactive pyrrole, they could be immobilized on the electrode surface by electrochemical copolymerization. This cyclic voltammetric scan could deposit different sequences of probe DNAs on designated electrodes, thus making a DNA array sensor. The target DNAs were detected using electrochemical stripping of silver, which was catalytically deposited on gold nanoparticles conjugated to the target DNA. The detection limit was well below 10^2^ cells/sample.

Microfluidic PCR chip is one of the earliest applications of microfluidic systems. To conduct PCR, it is essential to control a thermal cycle reproducibly in a chip. Currently, there are several different approaches for controlling temperature in the chip. Microfabricated electrodes are frequently used as heating elements because they can be easily implemented in the chip [38]. Portable thermal cycler that can accommodate the whole chip has been also developed [39]. Alternatively, infrared lamp has been used as a heating source [46,47]. The early development of a lab-on-a-chip sensor for bacterial detection traces back to late 1990's. Ramsey group demonstrated a glass microfluidic chip that could perform cell lysis, PCR, and electrophoresis [48]. In this early stage of development, heating elements were not incorporated within a glass chip. Thus, an off-chip thermal cycler was used for carrying out PCR. Then, electrophoresis was carried out to separate PCR products in the glass channel filled with gel polymer.

More elaborate forms of microfluidic chips containing on-chip heaters have been reported by Liu and Koh, respectively [49,50]. These lab chip sensors are equipped with valves and micropatterned electrodes in the chip, which made it easy to conduct on-chip PCR without contamination and evaporation problems. Photocrosslinkable gel and heat-sensitive wax have been used as valves in those systems. In the case of Koh's sensor, lysis was performed off-chip and then the cell lysate was introduced in the chip. The PCR product was separated using on-chip electrophoresis. Liu *et al.* performed cell lysis in a chip using thermal energy and then delivered the PCR product to an independent electrochemical sensing system for target DNA detection. Cady *et al.* reported an integrated microfluidic system for the detection of *Listeria monocytogenes* [45]. The microfluidic module chip was designed to mount on the top of a heater. A unique feature of this system lies in the on-chip purification of DNA before real-time PCR. Cell lysates were introduced in the microchannel that contained micropillar structures coated with silica. DNA could be selectively adhered on the pillar surface for rinsing. The sensor could detect ∼10^4^ cells/mL of *Listeria monocytogenes*.

In addition to fluorescence and electrical detection, a quartz crystal microbalance (QCM) has also been used as a sensitive pathogen sensor by capturing target cells on the sensor surface. Instead of detecting pathogen cells, Mao *et al.* focused on the detection of bacterial DNA using QCM [51]. Target bacterial DNAs amplified from off-chip PCR were hybridized with probe DNAs on the sensor surface. A change in the mass after hybridization could be amplified by conjugating nanoparticles to the target DNA. They demonstrated that they could sense *E. coli* O157:H7 as low as 2.7×10^2^ cells/mL.

#### Nucleic acid sensor without PCR

As mentioned previously, PCR-based pathogen sensor cannot provide the information of virulence of bacterial cells. To overcome the limitation of DNA-based sensor, several research groups have focused on RNA, because RNAs are easily decayed after cell death and the presence of RNA suggests the viability of bacterial cells. Dimov *et al.* have successfully demonstrated an integrated microdevice that can conduct RNA purification, nucleic acid sequence-based amplification (NASBA), and fluorescence detection in real-time [52]. NASBA is an amplification process that can produce a high copy number of RNA under isothermal (∼ 41 °C) conditions in an hour. The chip was constructed of multiple chambers and inlet ports. The bacterial cell lysate containing target RNA was purified in the first chamber containing silica beads and subsequently delivered to the second chamber for NASBA and fluorescence analysis. A real-time detection process took less than 30 min with the detection limit of 10^3^ cells/mL E. coli cells of crude lysate.

Several groups relied on 16S rRNAs for bacteria detection. Since several thousand copies of 16S rRNAs are contained in single cell, they could be detected without an amplification step [25,53]. DNA microarray has been used to detect 16S rRNAs. In addition to its multiplexing advantages it can be integrated with a microfluidic device [54]. Peplies *et al.* reported the detection of 16S rRNA extracted from bacteria in freshwater sediments [55]. Seventy different probe DNAs were spotted on the array for the test. The signal was obtained using both fluorescence in situ hybridization (FISH) and catalyzed reporter deposition methods (CARD). Previous research found a correlation between virulence of E. coli and a protein expression [56-58]. Thus, detecting genes that encodes the protein can discern the pathogenicity of certain E. coli cells. Basselet *et al.* developed an assay for assessing the pathogenicity of E. coli using electric DNA array chip [59]. This array technique will complement PCR-based sensor by providing essential information of bacterial pathogenicity.

### 2.3. Focusing strategies in microfluidic devices

In parallel with improving the sensitivity of a sensing probe, concentrating sample solution is a good strategy for enhancing the detection limit. Physical entrapment based on microstructure or microfilters, dielectrophoresis, and magnetic or polymeric beads were frequently employed to concentrate samples in a microfluidic channel. Lay *et al.* devised a microfluidic cell concentrator based on ultrafine (< 1 μm) microfilters fabricated on silicon wafer and demonstrated detection of bacterial cells using fluorescence-based immunoassay [33,60]. The system was designed to effectively reduce clogging and pressure drop that is frequently encountered in a microfluidic device having fine filter gaps. The *E. coli* cells were detected at the concentration of 10^6^ CFU/mL.

Rodriguez *et al.* developed a capillary electrophoretic method for the detection of bacteria in contaminated solution. The technique combined the effect of surfactants in the buffer solution and an injection spacer technique to focus the cells in a narrow zone. The method was capable of detection and identification of a spectrum of bacteria [61,62]. The electrophoresis sensor was further realized using PDMS based microfluidic chip for the detection of *E. coli* cells [63]. Ultrasound standing waves (USWs) were also used to focus the bacteria cells onto the sensor surface in a microfluidic system and the cells were detected using optical leaky waveguide [64]. In this system, the sensing chamber is only a quarter-wavelength long, thus allowing only one node to form to attract all the cells in the chamber. It is demonstrated that the USWs enhanced the capture of BG spores suspension on the chamber surface significantly, improving the detection limit by 100-fold. McGovern *et al.* demonstrated that a careful control of flow rate could differentiate specific binding of target bacterial spores to sensing surface among a mixture of other similar bacterial species by minimizing nonspecific binding [65,66]. Recently, Wu *et al.* reported a system combining a cantilever with a particle focusing method. The principle of concentrating target particles is based on alternating current electroosmosis particle focusing (ACEO) and they demonstrated that particles could be concentrated on the cantilever surface, which will help reduce incubation time with biological samples [67].

The Bashir group exploited dielectrophoresis to concentrate bacterial cells [37]. The microfluidic system was designed to utilize dielectrophoretic force combined with fluid flow to deviate all the cells into a small sensing channel for on-chip PCR and detection using interdigitated microelectrodes. The results demonstrated a three-fold increase in the signal through the preconcentration step. This paper shows that the sensor could detect as few as 60 cells of *L. monocytogenes* V7 in less than 90 min even in the presence of other bacterial strains.

## 3. Recent Sensing Strategies for Pathogen Detection Based on Nanomaterials

Current nanofabrication technology can make the size of a sensing probe comparable to those of bacteria or other target pathogens, improving the sensitivity and detection limit of a sensor enormously. In addition, the technology allows for an array of sensors, which can carry out high throughput detection. This chapter will cover recent progress of nanofabricated sensors for detecting pathogens.

### 3.1. Nanofabricated electrical sensors: nanowell, nanotube and nanowire

Nanotubes and nanowires have been used to construct miniaturized sensor probes due to their unique physical properties. The one dimensional structure of nanotubes and nanowires offer the smallest confinement for an electron transport along the longitudinal direction. Their large surface area promotes interaction between the target cells and nanomaterials, further improving the sensitivity. Towards the goal of developing nanotube or nanowire-based pathogen sensor, most of the earlier work has been focused on chemically modifying nanotubes to promote the solubility in aqueous solution and interaction with various biomolecules [68,69]. For example, Elkin *et al.* coated the carbon nanotube surfaces with bovine serum albumin (BSA) to improve the solubility of the nanotubes in aqueous solution, and further constructed nanotube-protein conjugates with pathogen-specific antibody. *E. coli* O157:H7 bound to the carbon nanotube was visualized using a confocal microscope [68]. Gu *et al.* functionalized the surface of single-walled carbon nanotubes with multivalent carbohydrate ligands to efficiently capture pathogenic *E. coli* cells [70,71]. In these specific cases, the galactose functionalities of the nanotube surfaces not only increases the solubility of the carbon nanotube, but also enhances interaction with receptors on pathogenic bacteria cells. These reports clearly show that functionalized carbon nanotubes can help identify, immobilize, and concentrate bacterial cells in a solution.

Direct measurement of conductance between two electrodes with a nano-sized gap can also be a highly sensitive technique for biosensing purpose. The applications have been extended to detect a pathogen cell bigger than small biomolecules, such as virus or antigens. Seo *et al.* fabricated a nanowell sensor with the gap of 150 nm between the two Ti electrodes [72]. The nanowell was used to detect the massive ion release from bacteria cells, which were infected by phage. The nano-sized gap between the two electrodes reduces the conducting path of electron transport, enabling noise analysis of the transport and enhancing sensitivity.

**Figure 3.**
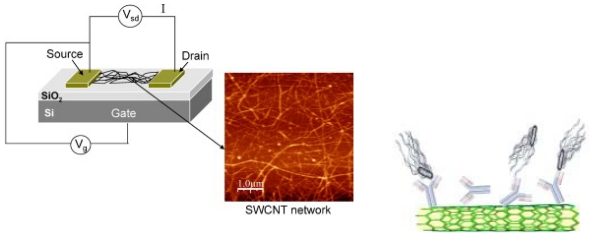
Experimental scheme for detecting *Salmonella Infantis* with a network of CNT-FETs functionalized with anti-Salmonella antibodies. Reproduced with permission from reference [73].

Several groups have demonstrated a high sensitive immunosensor based on a field effect transistor (FET) constructed with nanotubes. Villamizar *et al.* constructed a field effect transistor consisting of carbon nanotubes (CNT) for the detection of *Salmonella Infantis* [73]. They synthesized CNT networks on top of silicon dioxide to form CNT-FETs and functionalized the CNT networks with anti-*Salmonella* antibodies (See Figure 3). They showed that the sensor could detect 100 CFU/mL of cells in 1 hr. The sensor can also selectively detect the target bacterial cells in the presence of other strains of bacterial cells. A direct conductance measurement of nanobube-bacteria conjugates were reported by Suehiro *et al* [74]. In their work, carbon nanotubes were treated using microplasma to improve the solubility in aqueous solution. Using dielctrophoresis method carbon nanotube-bacterial conjugates were trapped into a small gap between two microelectrodes to form a bridge. The conductance between electrodes was monitored according to the concentration of *E. coli*. Using the same principle, peptide nanotubes coated with antibodies were also placed in the gap between two electrodes to detect viruses [75]. *Staphylococcus aureus* is one of the difficult bacteria to detect via direct immunoassay. The research group detected enterotoxins released from the bacteria instead of directly detecting the cells. The sensor measures a change in the capacitance between the electrodes upon the binding event of the pathogen.

Various nanowires have been incorporated into a sensing structure to improve the sensing limit. In an electrochemical sandwich biosensor, polyaniline nanowires were used as a molecular electrical transducer to report bacterial attachment [76]. The target bacteria cells were first bound to polyaniline-antibody conjugates. The complexes were then captured by the second antibodies immobilized on the surface between two electrodes. The polyaniline nanowires bound to the primary antibodies formed a conductance path between the two electrodes. The sensitivity of this sensor was found to be 10^1^ to 10^2^ CFU/mL of *B. cereus* pure cell culture. Mishra *et al.* fabricated a silicon nanowire transistor having 50 nm wide and connecting two gold pads having 150 nm in-between space [77]. Electrochemical impedance spectroscopy was used to detect enterotoxin in the range of 10–35 fM.

Nanowires consisting of multistriped bands of Ag and Au could form excellent platforms for multiplexed detection of pathogens [78]. Pathogen sensing relied on a sandwich immunoassay by utilizing antibodies immobilized on the nanowires. Fluorescence image showed the presence of a pathogen and optical reflectance image could identify the pathogen by the encoded stripe patterns of the nanowire. In this report, they could detect bacterial spores simulating *Bacillus anthracis* as low as 10^5^ CFU/mL.

### 3.2. Nanoparticles

Bio-conjugated nanoparticles emerged as a powerful tool for pathogen sensing by taking the advantage of easy control of size and functionalization. The nano-particles are used as a fluorescent label for detection or a conjugate label for signal amplification. Pascual *et al.* will review a detailed strategy for bacterial detection based on nanoparticles in this special issue of *Sensor*. Here, we will highlight a few significant examples of nanoparticle-based sensors recently reported.

#### Nanoparticles as biomarkers

**Figure 4.**
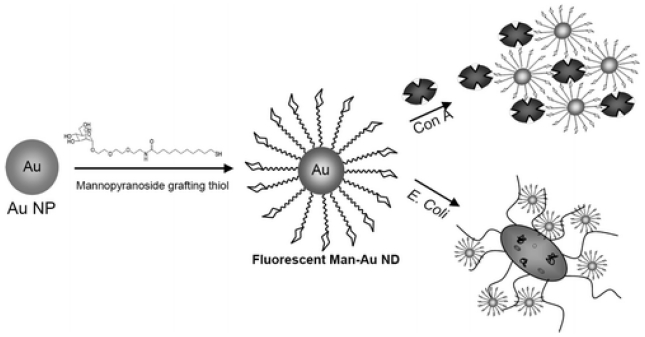
Schematic diagram showing the preparation steps for fluorescent carbohydrate-Au nanoparticles for the detection of *E. coli*. Reproduced with permission from reference [82].

Semiconductor quantum dots (QDs) exhibit many advantages over traditional organic dye molecules in fluorescent labelling. The QDs show narrower emission peaks, higher emission intensity, and longer life time than dye molecules. Over the years, various QDs has been functionalized to form QD-antibody conjugates and then used as a fluorescence maker for pathogen detection [79]. Yang and Li used two different sets of QD-antibody conjugates to detect two different strains of bacteria, i.e. *S. Typhimurium* cells and *E. coli* O157:H7 cells [80]. First, magnetic beads coated with antibodies were used to capture bacteria cells. Then, QD-antibody conjugates were introduced to visualize the attached bacterial cells. Two fluorescence peaks were recognized at different wavelengths resulting from two different QD labels. The detection limit was 10^4^ CFU/mL and the whole process could be completed within 2 hours. Liu *et al.* used a flowing chamber containing a microporous filter for detection [81]. The filter consisted of a microporous polymer membrane and served as a detection matrix. The probe antibodies were immobilized on the surface of the filter membrane to capture target bacteria cells. The QD-antibody conjugates labeled the captured cells. This sensor can detect *E. coli* O157:H7 cells as low as 2.3 CFU/mL. Utilizing surface plasmon effects, Huang *et al.* developed a method that relies on carbohydrate-protected Au nanoparticles for bacterial detection. The method is relatively simple and capable of sensing both lectins and bacteria [82] (See Figure 4).

Various new strategies were proposed to utilize nanoparticles for enhancing detection limits. Zhao and coworker synthesized silica nanoparticles doped with fluorescence dye molecules and chemically modified their surfaces for rapid bioassay [83]. Many bio-conjugated nanoparticles could be attached to a single bacterium cell. Because each silica particle encapsulates thousands of fluorescence dye molecules, it shows a remarkable high fluorescence signal. The assay was highly sensitive for rapid detection of single bacterium. Edgar *et al.* reported a highly sensitive and rapid phage-based detection. The method utilizes engineered host-specific bacteriophage to form phage and QDs conjugation. The method presented a detection of 10 cells/mL in 1 hour with a 100-fold amplification of the signal over background [84].

#### Nanoparticles as a signal amplifier

One of the main applications of immuno-conjugated nanoparticles is to use them as a matrix to immobilize and concentrate target pathogen cells. Magnetic beads, for example, can be easily manipulated using magnetic fields and detected with various methods. Naja *et al.* used magnetic immuno-nanoparticles to capture *E. coli* cells and detect the bacteria via a UV resonance Raman method [85].

Farrell *et al.* developed an assay using magnetic beads for the detection of *B. anthracis* spore stimulant. The *B. globigii* spores were captured by the beads modified with specific antibodies. Then, a fluorescence signal was obtained from the enzyme reaction catalyzed by alkaline phosphatase enzyme conjugated to the secondary antibody [86]. Similar methods were used to detect *E. coli* [87]. Mujika *et al.* developed a giant magnetoresistance (GMR) sensor for the detection of *E. coli* O157: H7 [9]. The sensor surface was functionalized with specific antibodies to capture the pathogen cells under flow conditions. The magnetic particles bearing antibodies were conjugated to the cells to form a sandwich structure. The GMR sensor detects a change in the straight field generated by the attached magnetic beads.

Lin *et al.* improved the detection limit of *E. coli* O157:H7 by employing gold nanoparticles for amperometric immunoassay [88]. They detected bacterial cells using a sandwich immunoassay. After cells were attached to the primary antibody immobilized on the electrode surface, secondary antibodies tagged with peroxidase enzymes were conjugated to the cells. The oxidation current of hydrogen peroxide was measured as a signal. The presence of gold nanoparticles tagged with ferrocene molecules on the electrode surface greatly contributed to the amplification of the current signal. The gold nanoparticles enhance not only the surface area of electrodes, but also the rate of electron transfer between peroxidase enzymes and electrodes. In addition, ferrocene was used as a mediator for electrochemical reaction between hydrogen peroxide and peroxidase enzyme. The sensor worked at the concentration range of 10^2^–10^7^ CFU/mL.

## 4. Conclusions and Prospects

In this review, we covered recent progress in the pathogen sensors that exploit micro- and nano-fabrication technology. Fully integrated and automated lab-on-a-chip sensor will be an ultimate platform for on-site pathogen detection whether the sensing relies on immuno-reaction or nucleic acid hybridization. Most recent work on nanoscaled pathogen sensors based on nanotubes, nanowires, and nanowells reveal a potential for high sensitivity detection despite their infancy in pathogen sensing. However, the challenge is to make them an off-the-shelf device available for routine use in the food industry and environmental monitoring.

With the maturity of the current nanofabrication technology, the fabrication and assembling of the new sensors has become a straightforward process. However, reliability, repeatability, durability, and ease-of operation are important issues for lab-on-a-chip pathogen sensors. For example, a common issue in microfluidic devices is a clogging problem caused by non-selective binding that significantly reduces the reliability and life time of the sensor. In addition, despite the progress in the development of new types of valves, the integration of a complex process within a single chip still faces challenges. Precise control of metering and transporting solution is important to minimize potential contamination between steps.

In our view, extremely-sensitive immunosensors will be suitable for fast screening in terms of response time and sensitivity. The result of fast screening may be further confirmed by PCR or other nucleic-acid based detection. This combined analysis will not take as much time as cell culture and plating methods. Several portable analytical systems based on detecting nucleic acids have been already commercialized by Cepheid, Gen-Probe, Idaho Technology, IQuum, and several other companies.
